# Prevalence, trends, and factors associated with hypertensive crisis among Peruvian adults

**DOI:** 10.1590/0102-311XEN155123

**Published:** 2024-02-19

**Authors:** Victor Calderon-Ocon, Fiorella Cueva-Peredo, Antonio Bernabe-Ortiz

**Affiliations:** 1 Universidad Científica del Sur, Lima, Perú.

**Keywords:** Hypertension, Hypertensive Crisis, Prevalence, Health Survey, Hipertensión, Crisis Hipertensiva, Prevalencia, Encuestas Epidemiológicas, Hipertensão, Crise Hipertensiva, Prevalência, Inquéritos Epidemiológicos

## Abstract

There are few studies focused on the epidemiology of hypertensive crisis at the population level in resource-constrained settings. This study aimed to determine the prevalence and trends over time of hypertensive crisis, as well as the factors associated with this condition among adults. A secondary data analysis was carried out using the *Peruvian Demographic and Family Health Survey* (ENDES). Hypertensive crisis was defined based on the presence of systolic (≥ 180mmHg) or diastolic (≥ 110mmHg) blood pressure, regardless of previous diagnosis or medication use. The factors associated with our outcome were evaluated using multinomial logistic regression, and the trend of hypertensive crisis was evaluated using the Cochrane-Armitage test. Data from 260,167 participants were analyzed, with a mean age of 44.2 (SD: 16.9) years and 55.5% were women. Hypertension prevalence was 23% (95%CI: 22.7-23.4) and, among them, 5.7% (95%CI: 5.4-5.9) had hypertensive crisis, with an overall prevalence of 1.5% (95%CI: 1.4-1.6). From 2014 to 2022, a significant decrease in the prevalence of hypertensive crisis was observed, from 1.7% in 2014 to 1.4% in 2022 (p = 0.001). In the multivariable model, males, increasing age, living in urban areas, high body mass index, and self-reported type 2 diabetes were positively associated with hypertensive crisis, whereas higher educational level, socioeconomic status, and high altitude were inversely associated. There is a need to improve strategies for the diagnosis, treatment, and control of hypertension, especially hypertensive crisis.

## Abstract (es)

Pocos estudios se han centrado en la epidemiología de la crisis hipertensiva a nivel poblacional en entornos de recursos limitados. El objetivo de este estudio fue determinar la prevalencia y tendencia, a lo largo del tiempo, de la crisis hipertensiva y los factores asociados a esta condición en adultos. Se realizó un análisis de datos secundarios utilizando la *Encuesta Demográfica de Salud Familiar* (ENDES) de Perú. La crisis hipertensiva se definió en función de la presencia de presión arterial sistólica (≥ 180mmHg) o diastólica (≥ 110mmHg), independientemente del diagnóstico previo o del uso de medicamentos. Los factores asociados a los resultados se evaluaron mediante regresión logística multinomial, y la tendencia a la crisis hipertensiva se estimó mediante la prueba Cochran-Armitage. Los datos de 260.167 participantes, con una media de 44,2 años (DE: 16,9) y 55,5% mujeres, fueron analizados. La prevalencia de hipertensión fue del 23% (IC95%: 22,7-23,4), de la cual el 5,7% (IC95%: 5,4-5,9) tuvo crisis hipertensiva, con una prevalencia general del 1,5% (IC95%: 1,4-1,6). En el período 2014-2022 se constató una disminución significativa en la prevalencia de crisis hipertensiva, del 1,7% en 2014 al 1,4% en 2022 (p = 0,001). En el modelo multivariable, el sexo masculino, el aumento de la edad, vivir en áreas urbanas, el alto índice de masa corporal y la diabetes autoinformada se asociaron positivamente con la crisis hipertensiva, mientras que mayor nivel educativo, nivel socioeconómico y elevada altitud estuvieron asociadas de manera inversa. Es necesario mejorar las estrategias para el diagnóstico, el tratamiento y el control de la hipertensión, especialmente de la crisis hipertensiva.

## Abstract (pt)

Há poucos estudos focados na epidemiologia da crise hipertensiva em nível populacional em ambientes com recursos limitados. O objetivo deste estudo foi determinar a prevalência e a tendência, ao longo do tempo, da crise hipertensiva e fatores associados a essa condição em adultos. Uma análise de dados secundários foi realizada usando a *Pesquisa Demográfica de Saúde Familiar* (ENDES) do Peru. Crise hipertensiva foi definida com base na presença de pressão arterial sistólica (≥ 180mmHg) ou diastólica (≥ 110mmHg), independentemente de diagnóstico prévio ou uso de medicação. Os fatores associados aos resultados foram avaliados por meio de regressão logística multinomial, e a tendência de crise hipertensiva foi avaliada pelo teste de Cochrane-Armitage. Os dados de 260.167 participantes, com média de 44,2 anos (DP: 16,9) e 55,5% mulheres, foram analisados. A prevalência de hipertensão foi de 23% (IC95%: 22,7-23,4), dentre eles, 5,7% (IC95%: 5,4-5,9) apresentaram crise hipertensiva, com prevalência geral de 1,5% (IC95%: 1,4-1,6). De 2014 a 2022, observou-se queda significativa na prevalência de crise hipertensiva, de 1,7% em 2014 para 1,4% em 2022 (p = 0,001). No modelo multivariável, sexo masculino, idade crescente, residir em área urbana, índice de massa corporal elevado e diabetes autorreferido associaram-se positivamente à crise hipertensiva, enquanto maior escolaridade, nível socioeconômico e altitude elevada associaram-se inversamente. Há necessidade de aprimorar as estratégias de diagnóstico, tratamento e controle da hipertensão arterial, especialmente da crise hipertensiva.

## Introduction

Hypertension is one of the most relevant risk factors for cardiovascular disease worldwide, being the leading cause of preventable disability and mortality worldwide ^1^, and responsible of more than 9 million deaths per year ^2^. Approximately 32% and 34% of women and men, respectively, aged 30 to 70 years, have this condition ^3^, whereas these estimates vary from 12% to 37% in Latin America, according to data collected by the Latin America Society of Hypertension (LASH) ^4^. In Peru, one out of every five people has hypertension ^5^; less than 50% are aware of their condition, and only one out of 20 Peruvians with hypertension maintain controlled blood pressure ^6^, predisposing them to the development of hypertensive crisis.

Hypertensive crisis is defined as the presence of systolic blood pressure (SBP) ≥ 180mmHg or diastolic blood pressure (DBP) ≥ 110mmHg ^7^, regardless of a previous hypertension diagnosis. Hypertensive crises are of two types, according to clinical management: hypertensive emergency, characterized by target organ damage, and hypertensive urgency, without such damage ^8^. Hypertensive crisis is an event that arises due to the failure of regulatory mechanisms ^9^, and increased endogenous vasoconstrictors due to endothelial damage secondary to hypertension ^10^, in the context of risk factors such as sedentary lifestyle, smoking, and poor adherence to antihypertensive treatment, which causes systemic and organ-specific complications. Hypertensive emergency is one of the most dangerous events, with target organ damage, especially in the central nervous system (e.g., stroke, hypertensive encephalopathy, etc.), but also in other organs (e.g., acute myocardial infarction, aortic dissection, etc.), which, in addition to requiring individualized management ^9^, is associated with significant mortality rates.

According to the literature, approximately 1% of individuals experience a hypertensive crisis (i.e., hypertensive urgency or emergency) at some point in their lives ^11^. In contrast, the prevalence of hypertensive crises among the hypertensive population may be up to 2%; however, 23% of hypertensive crises occur in people who have not previously been diagnosed with hypertension ^9^. In addition, women are more likely to develop hypertensive crises, and this condition is more frequent among those with poor adherence to antihypertensive treatment. However, other studies report statistically higher prevalence among men ^12^. Thus, characterizing subjects with hypertensive crisis is essential to understand the epidemiology of this condition in resource-constrained settings.

Therefore, this study aimed to determine the prevalence and trends of hypertensive crisis over time, as well as the sociodemographic and behavioral factors associated with this condition among Peruvian adults.

## Materials and methods

### Study design

A secondary data analysis was conducted using information from the *Peruvian Demographic and Family Health Survey* (ENDES, acronym in Spanish). The ENDES is a cross-sectional, population-based, and nationally representative survey, conducted annually, that collects information from the 25 Peruvian regions ^13^. For this analysis, information from the Health Questionnaire, including information on blood pressure, from 2014 to 2022, was merged and analyzed.

### Study population and sampling

ENDES holds a two-stage, probabilistic, balanced, independent sampling, stratified by regions and areas (i.e., rural and urban), with a similar methodology but different framework used for the selected years. The ENDES target population comprises households members, habitual residents, and those who spent the night before the day of the interview in the selected household. In addition, ENDES includes all women aged 15 to 49 years and children under 5 years in the selected household ^13^.

For this study, all individuals aged ≥ 18 years were included in the analysis. Pregnant and lactating women, as well as those without information on blood pressure measurement, were excluded.

By combining several databases from different years, a statistical power over 90% was achieved to find a 0.5% difference between the levels of most of our covariates (e.g., male vs. female), comparing hypertensive crisis vs. normotensive subjects. This statistical power assumed a 5% confidence level and a 1.5 design effect as previously described for similar studies in our context ^14^.

### Definition of variables

The dependent variable in the study was hypertensive status, divided into two categories: (a) subjects with normal blood pressure (normotensive), defined as those with SBP < 140mmHg, DBP < 90mmHg, and no previous diagnosis of hypertension or use of anti-hypertensive medication; and (b) subjects with hypertension, defined as those with SBP ≥ 140mmHg, or DBP ≥ 90mmHg, or previous medical diagnosis of hypertension or self-reported use of antihypertensive medication ^15^. As our analysis focused mainly on hypertensive crisis, the hypertensive category was further divided into two groups: those with isolated hypertension and those who had hypertensive crisis at the time of evaluation, defined according to international guidelines as the presence of SBP ≥ 180mmHg or DBP ≥ 110mmHg ^7^, regardless of previous diagnosis or medication use. It was not possible to include organ damage, as this variable was not present in the database used.

Given the exploratory nature of the analysis, our covariates included different potential factors associated with hypertension. Sociodemographic variables include sex (male or female); age (< 40, 40-59, and 60+ years); educational level (< 7, 7-11, and 12+ years); area (rural or urban); altitude, measured in meters above sea level (m.a.s.l.) and categorized into three groups (< 500, 500-2,499, and 2,500+); and socioeconomic status, defined by a wealth index, a composite measure of the household’s cumulative living standard. The wealth index was calculated using easy-to-collect information on a household’s ownership of selected assets (i.e., television, bicycle, car, etc.), materials used for housing construction (concrete, cement, wood, etc.), and type of access to water or sanitation services. To do this, we used the *Demographic and Health Surveys* (DHS) program approach ^16^ and divided the numerical value obtained into tertiles for analysis (low, middle, and high). In addition, lifestyle-related and anthropometric variables were also included, such as smoking, assessed by self-reported tobacco consumption in the 30 days prior to the survey (non-smoker or smoker); alcohol use, assessed by self-reported alcohol consumption in the 30 days prior to the interview (non-drinker or drinker); body mass index (BMI), obtained using weight and height information according to international guidelines and categorized as normal (BMI < 25kg/m^2^), overweight (25 to < 30kg/m^2^), and obese (≥ 30kg/m^2^); and type 2 diabetes, based on self-reported previous medical diagnosis.

### Procedures

Data collection was carried out using paper-based questionnaires and personal digital assistant devices in 2014 and 2015 surveys, but using a mobile device (tablet computer) from 2016 onwards. All questionnaires and anthropometric evaluations are usually carried out with direct interviews (face-to-face approach) by trained personnel who visited selected households. However, during the COVID-19 pandemic (2020 and 2021 surveys), an important proportion of the questionnaires were administered by telephone, whereas blood pressure measurements were taken after the mandatory social isolation enforced by the Peruvian government.

Different strategies were implemented to guarantee adequate blood pressure measurements. Thus, at least 30 minutes of rest were given if the interviewee had smoked, consumed coffee, tea, alcohol, or other beverages before the interview. Otherwise, the interviewee was asked to sit down and rest for at least five minutes before the first blood pressure measurement, which was determined using a digital blood pressure monitor (OMRON, HEM-7113, https://www.omron.com), placed on the right arm. Blood pressure was measured twice, with the second assessment taking place two minutes apart from the first one. The average of both values was used for analysis. Finally, BMI was calculated based on weight, measured with the person standing on a standard scale, and height, measured with a multi-purpose mobile stadiometer, both taken by trained personnel.

### Statistical analysis

Statistical analysis was carried out using Stata 16.0 (https://www.stata.com), considering the multistage sampling of the survey, and the appropriate subcommands for handling subpopulations ^17^. Initially, the study population was described using means and standard deviation (SD) for numeric variables, and frequencies and proportions for categorical variables. The sociodemographic and behavioral characteristics of the study population were tabulated using the chi-square test with Rao-Scott second-order correction ^18^. The prevalence of individuals with hypertension and hypertensive crises was estimated and reported with their respective 95% confidence intervals (95%CI). Moreover, the Cochrane-Armitage trend test was used to evaluate changes in hypertensive crisis over time.

Finally, given the exploratory nature of this study, multinomial logistic regression models were constructed to determine the factors independently associated with hypertension and hypertensive crisis compared to normotensive population. For this reason, all variables were evaluated using a bivariable approach and then, regardless of statistical significance, were included in the multivariable model, reporting the odds ratios (OR) and 95%CI. For all models, a p-value < 0.05 was considered statistically significant. As a sensitivity analysis, the models were run again, but excluding those previously diagnosed with hypertension, to focus on those with no previous diagnosis.

### Ethics

This study was approved by the Research Ethics Committee of the Scientific University of the South (Universidad Científica del Sur, Peru; code: 004-2021-PRE15). The data used for the analyses are freely available, and records are de-identified to guarantee participants’ anonymity.

## Results

### Characteristics of the study population

A total of 328,167 participants responded to the ENDES Health Questionnaire from 2014 to 2022; however, 17,331 records were excluded for being < 18 years; 4,003 for being pregnant women or lactating, and 46,666 for not having data on blood pressure. Thus, the final sample for analysis was 260,167 participants (79.3% of the initial sample), with a mean age of 44.2 (SD: 16.9) years, 54.5% were women, and 76.2% dwelled in urban areas.

### Prevalence, trends, and associated factors of hypertension and hypertensive crisis

A total of 46,646 individuals (23%; 95%CI: 22.7-23.4) had hypertension and, among this group, 2,638 subjects (5.7%; 95%CI: 5.4-5.9) had hypertensive crisis. Thus, the overall prevalence of hypertensive crisis in the total study population was 1.5% (95%CI: 1.4-1.6).

From 2014 to 2022, an increase in the prevalence of hypertension was found, from 22.9% to 25.2% (p-value for trend < 0.001), whereas a significant decrease in the prevalence of hypertensive crisis was observed, from 1.7% in 2014 to 1.4% in 2022 (p-value for trend = 0.001). In addition, the prevalence of hypertensive crisis was more frequent among those without a previous diagnosis of hypertension compared to those with a previous diagnosis (7.7% vs. 5.4%; p < 0.001).

### Description of the population according to their hypertensive status

In the bivariable analysis (Table 1), those with hypertensive crisis were predominantly females (p < 0.001), aged over 60 (p < 0.001), had a lower level of education (p < 0.001), high socioeconomic status, and came from urban areas (p < 0.001) and low-altitude sites (p < 0.001). Besides, those with hypertensive crisis reported lower smoking (p < 0.001) and alcohol use (p < 0.001). Finally, hypertensive crisis was more frequent among those with obesity and those with type 2 diabetes (p < 0.001).

**Table 1:** Description of the adult population (≥ 18 years old) comparing normotensive, hypertensive, and hypertensive crisis subjects. Peru, 2014-2022.

Characteristics	Hypertensive status (n (%))			p-value
	Normal (n = 213,533)	Hypertension (n = 43,997)	Hypertensive crisis (n = 2,637)	
Sex				< 0.001
Female	122,742 (56.0)	21,692 (49.3)	1,385 (52.3)	
Male	90,791 (44.0)	22,305 (50.7)	1,252 (47.7)	
Age (years)				< 0.001
< 40	134,703 (55.1)	10,544 (18.1)	150 (4.2)	
40-59	56,958 (31.4)	15,745 (36.0)	633 (24.4)	
60+	21,872 (13.5)	17,708 (45.9)	1,854 (71.4)	
Educational level (years)				< 0.001
< 7	53,033 (23.4)	15,265 (32.9)	1,136 (45.6)	
7-11	90,185 (41.4)	14,528 (37.4)	638 (33.6)	
12+	63,540 (35.2)	10,288 (29.7)	393 (20.8)	
Socioeconomic status				< 0.001
Low	72,957 (25.4)	14,044 (21.3)	1,003 (25.8)	
Middle	70,208 (30.4)	13,721 (30.1)	703 (27.9)	
High	70,395 (44.2)	16,243 (48.6)	932 (46.4)	
Area				< 0.001
Urban	138,178 (75.2)	29,336 (79.5)	1,695 (78.8)	
Rural	75,382 (24.8)	14,672 (20.5)	943 (21.2)	
Altitude (m.a.s.l.)				< 0.001
< 500	102,994 (59.9)	22,797 (65.9)	1,390 (67.9)	
500-2,499	43,738 (15.7)	8,709 (14.3)	535 (14.3)	
2,500+	66,828 (24.4)	12,502 (19.8)	713 (17.8)	
Smoking				< 0.001
Non-smoker	191,288 (89.1)	39,425 (90.2)	2,459 (94.2)	
Smoker	22,154 (10.9)	4,555 (9.8)	176 (5.8)	
Alcohol use				< 0.001
Non-drinker	143,311 (64.3)	30,445 (68.8)	2,015 (75.9)	
Drinker	70,099 (35.7)	13,497 (31.2)	616 (24.1)	
BMI				< 0.001
Normal	83,067 (38.3)	10,977 (22.5)	769 (25.5)	
Overweight	86,058 (40.9)	17,538 (41.0)	996 (40.3)	
Obesity	43,678 (20.8)	15,139 (36.5)	831 (34.2)	
Type 2 diabetes				< 0.001
No	184,153 (97.4)	35,313 (89.6)	2,123 (89.3)	
Yes	3,561 (2.6)	3,408 (10.4)	240 (10.7)	

BMI: body mass index; m.a.s.l.: meters above sea level.

Note: columns may not add due to missing data - sex (n = 39), age (n = 39), educational level (n = 11,200), smoking (n = 149), alcohol use (n = 223), BMI (n = 1,153), and type 2 diabetes (n = 31,408). Proportions were estimated without considering missing values.

### Factors associated with hypertensive crisis

In the multiple multinomial regression model (Table 2), males had a higher prevalence of hypertensive crisis compared to females. In addition, increasing age was associated with a higher prevalence of hypertensive crisis, especially among those aged over 60. Higher educational level and socioeconomic status were associated with a lower prevalence of hypertensive crisis. Subjects from urban areas had a higher prevalence of hypertensive crisis, whereas higher altitude showed an inverse relationship with the outcome of interest. Finally, both BMI and self-reported diabetes were associated with a higher prevalence of hypertensive crisis.

**Table 2:** Factors associated with hypertension and hypertensive crisis in adults (≥ 18 years old): simple and multiple multinomial models. Peru, 2014-2022.

Characteristics	Bivariable model		Multivariable model	
	Hypertension	Hypertensive crisis	Hypertension	Hypertensive crisis
	OR (95%CI)	OR (95%CI)	OR (95%CI)	OR (95%CI)
Sex (vs. female)				
Male	1.31 (1.26-1.35)	1.16 (1.03-1.31)	1.57 (1.50-1.64)	1.67 (1.44-1.96)
Age (years) (vs. < 40)				
40-59	3.49 (3.33-3.66)	10.16 (7.57-13.64)	2.93 (2.78-3.08)	8.26 (6.04-11.28)
60+	10.36 (9.88-10.87)	69.18 (52.55-91.07)	9.49 (8.95-10.06)	56.70 (41.88-76.76)
Educational level years (vs. < 7)				
7-11 years	0.64 (0.62-0.67)	0.42 (0.35-0.49)	0.94 (0.89-0.99)	0.79 (0.66-0.96)
12+	0.60 (0.57-0.63)	0.30 (0.25-0.36)	0.89 (0.83-0.94)	0.59 (0.47-0.74)
Socioeconomic status (vs. low)				
Middle	1.18 (1.13-1.23)	0.91 (0.78-1.05)	1.06 (0.99-1.12)	0.81 (0.66-0.98)
High	1.31 (1.26-1.37)	1.04 (0.91-1.19)	1.01 (0.95-1.08)	0.76 (0.62-0.94)
Area (vs. rural)				
Urban	1.28 (1.24-1.33)	1.23 (1.10-1.38)	1.19 (1.13-1.26)	1.55 (1.29-1.86)
Altitude (m.a.s.l.) (vs. < 500)				
500-2,499	0.82 (0.79-0.86)	0.80 (0.70-0.93)	0.91 (0.86-0.96)	0.81 (0.68-0.96)
2,500+	0.74 (0.71-0.77)	0.64 (0.56-0.73)	0.85 (0.81-0.89)	0.55 (0.47-0.66)
Smoking (vs. non-smoker)				
Smoker	0.89 (0.84-0.94)	0.50 (0.40-0.64)	1.09 (1.01-1.17)	0.89 (0.67-1.17)
Alcohol use (vs. non-drinker)				
Drinker	0.82 (0.79-0.85)	0.57 (0.50-0.66)	0.99 (0.95-1.04)	0.95 (0.80-1.12)
BMI (vs. normal)				
Overweight	1.70 (1.63-1.78)	1.47 (1.28-1.70)	1.73 (1.64-1.82)	1.57 (1.31-1.88)
Obesity	2.97 (2.85-3.11)	2.45 (2.11-2.85)	3.13 (2.95-3.32)	2.97 (2.45-3.60)
Type 2 diabetes (vs. no)				
Yes	4.38 (4.05-4.75)	4.50 (3.63-5.58)	2.18 (1.98-2.40)	1.79 (1.41-2.82)

95%CI: 95% confidence interval; BMI: body mass index; m.a.s.l.: meters above sea level; OR: odds ratio.

The sensitivity analysis including only those with a previous diagnosis of hypertension showed quite similar results (Table 3). Of note, self-reported diabetes was no longer significant, whereas some categories of educational level, socioeconomic status, and altitude also lost their significance.

**Table 3:** Factors associated with hypertension and hypertensive crisis among adults (≥ 18 years old) with no previous diagnosis of hypertension: simple and multiple multinomial models. Peru, 2014-2022.

Characteristics	Bivariable model		Multivariable model	
	Hypertension	Hypertensive crisis	Hypertension	Hypertensive crisis
	OR (95%CI)	OR (95%CI)	OR (95%CI)	OR (95%CI)
Sex (vs. female)				
Male	2.26 (2.16-2.37)	1.71 (1.43-2.06)	2.76 (2.60-2.93)	2.48 (1.95-3.16)
Age (years) (vs. < 40)				
40-59	2.81 (2.65-2.98)	7.47 (5.12-10.90)	2.49 (2.33-2.65)	6.84 (4.58-10.21)
60+	6.29 (5.92-6.69)	43.31 (30.60-61.29)	6.09 (5.64-6.57)	39.40 (26.78-57.97)
Educational level years (vs. < 7)				
7-11 years	0.77 (0.73-0.82)	0.47 (0.37-0.60)	0.93 (0.87-1.00)	0.87 (0.66-1.14)
12+	0.68 (0.64-0.73)	0.30 (0.22-0.40)	0.85 (0.79-0.93)	0.56 (0.40-0.79)
Socioeconomic status (vs. low)				
Middle	1.15 (1.09-1.21)	0.79 (0.63-0.98)	1.04 (0.97-1.13)	0.85 (0.65-1.11)
High	1.16 (1.10-1.22)	0.70 (0.57-0.85)	0.91 (0.84-0.99)	0.64 (0.47-0.87)
Area (vs. rural)				
Urban	1.24 (1.19-1.30)	0.97 (0.82-1.14)	1.27 (1.18-1.36)	1.72 (1.34-2.22)
Altitude (m.a.s.l.) (vs. < 500)				
500-2,499	0.80 (0.76-0.85)	0.86 (0.70-1.07)	0.85 (0.79-0.92)	0.79 (0.61-1.01)
2,500+	0.74 (0.70-0.77)	0.85 (0.70-1.03)	0.79 (0.75-0.85)	0.57 (0.45-0.73)
Smoking (vs. non-smoker)				
Smoker	1.19 (1.11-1.28)	0.67 (0.49-0.91)	1.10 (1.01-1.20)	0.94 (0.66-1.34)
Alcohol use (vs. non-drinker)				
Drinker	1.03 (0.98-1.08)	0.65 (0.52-0.80)	1.03 (0.97-1.10)	0.92 (0.66-1.34)
BMI (vs. normal)				
Overweight	1.68 (1.59-1.77)	1.19 (0.97-1.47)	1.80 (1.67-1.92)	1.38 (1.06-1.78)
Obesity	2.75 (2.59-2.92)	1.66 (1.33-2.08)	3.30 (3.05-3.56)	2.37 (1.78-3.16)
Type 2 diabetes (vs. no)				
Yes	2.09 (1.84-2.37)	1.53 (0.92-2.54)	1.10 (0.95-1.28)	0.65 (0.37-1.14)

95%CI: 95% confidence interval; BMI: body mass index; m.a.s.l.: meters above sea level; OR: odds ratio.

## Discussion

According to our results, approximately 1.5% of the total number of individuals evaluated, but 5% of the total number of cases previously diagnosed with hypertension, had a hypertensive crisis. Additionally, whereas hypertension prevalence has increased over time, there was a reduction in the prevalence of hypertensive crisis during the studied period. In the multiple multinomial regression model, being male, older age, especially those over 60, living in an urban area, having overweight/obesity, and having type 2 diabetes were positively associated with hypertensive crisis. On the other hand, higher educational level, higher socioeconomic status, and high altitude, especially living 2,500 m.a.s.l., were inversely associated with hypertensive crisis. Our findings were quite similar in the sensitivity analysis including those with no previous diagnosis of hypertension.

A relatively recent systematic review, including eight observational studies, reported that the prevalence of hypertensive crisis was 1.2% ^11^, varying from 0.3% for hypertensive emergency to 0.9% for hypertensive urgency; however, this review only included studies carried out in emergency departments. Another study in Brazil, evaluating 508 individuals in emergency departments, reported a 0.6% prevalence of hypertensive crisis, but a case was defined based only on DBP ≥ 120mmHg ^19^. Despite the different approaches, our analysis reported similar estimates using population-based data.

In a prospective study that enrolled 7,600 outpatients from a medical center in Tanzania, a 2.6% prevalence of hypertensive crisis was reported ^20^, an estimate more than twice as high the one reported in this study. Nevertheless, the mean age in the latter report was 62 years, much higher than in our population-based study, due to the fact that older age increases the cardiovascular risk, including hypertensive disorders ^21^. A Canadian study carried out in an urban center using a mobile clinic evaluated a total of 1,097 subjects of a wider age range (16-92 years), and the result was a 2% prevalence among patients without symptoms ^22^. In this latter analysis, the prevalence of hypertension was 50%, highlighting the presence of selection bias, i.e., those individuals whose researchers suspected of hypertension were evaluated in the mobile clinic, leading to other associated problems, such as poor adherence to treatment ^23^.

Our findings also show that approximately 5% of people with hypertension have had a hypertensive crisis, an estimate above the world statistics ^24^, despite the fact that the frequency of hypertension has been dropping over time due to better access to and use of antihypertensive drugs ^3^ ^,^ ^25^. Our results, however, suggest a lack of adequate blood pressure control, as demonstrated in a previous study ^6^, with the subsequent risk of presenting a hypertensive crisis. Additionally, the prevalence of hypertensive crisis differed when estimated in subjects without a diagnosis of hypertension compared to those with a previous diagnosis. The prevalence of hypertensive crisis among subjects with no history of hypertension was much lower than that found in an Italian study (23%) ^12^ including individuals in an emergency room and, obviously, much lower compared to a study including cases of hypertensive crisis with target organ damage, where more than half had no previous diagnosis of hypertension ^23^. Nevertheless, the highest proportion of hypertensive crises occurs among those with a history of hypertension ^26^. Finally, the prevalence of hypertension reported in this study was similar to that reported in previous studies ^6^, but below the 40% expected for Latin America ^27^.

Age increases the risk of developing hypertension and, therefore, hypertensive crisis, which is explained from a physiological perspective as aging generates endothelial changes and collagen deposition at the arterial level ^21^. Regarding sex, our results differ from a previous study describing hypertensive crises in emergency rooms ^26^. Additionally, the higher prevalence of hypertensive crises in women in the latter manuscript may be secondary to menopause ^28^ or to better health access or concern about health among women. Smoking and alcohol use were not associated with hypertensive crisis in our study, but both were associated with hypertensive crisis in a prospective study carried out in Tanzania ^29^.

BMI, especially obesity, was a factor associated with hypertensive crisis, a common finding in other Latin American countries, such as Brazil ^30^, mainly due to the lack of physical activity and the presence of dyslipidemia, which were not evaluated in our study. Finally, self-reported type 2 diabetes was also associated with hypertensive crisis, since diabetes affects the vascular endothelium ^19^, with a subsequent increase in cardiovascular risk.

Worldwide, there are several guidelines for diagnosing and treating hypertension, such as those of the American College of Cardiology/American Heart Association of 2017 ^8^, or that of the European Society of Cardiology of 2018 ^7^, which mention these complications and appropriate management. In Peru, although there are some guidelines developed by Social Security (EsSalud, acronym in Spanish) and the Peruvian Ministry of Health (MINSA, acronym in Spanish), only the definition of hypertensive crisis is mentioned with referral to a more complex healthcare facility. This is because these guidelines focus on detection and diagnosis, rather than proper care and management.

Our results highlight the need to adequately manage hypertension and hypertensive crisis, both of which are associated with cardiovascular events and mortality ^31^. The deficiencies in the Peruvian healthcare system are well recognized, especially since most first-, second-, and third level healthcare facilities do not hold adequate capacity, including infrastructure, equipment, and supplies, affecting their management level ^32^. These conditions may affect the quality of care, especially in the prevention and management of cases with organ damage. Therefore, our study expands current knowledge by presenting estimates of the hypertensive crisis at the population level in Peru, in a context where hypertension awareness, treatment, and control rates are poor ^6^.

This study analyzed consecutive years of ENDES data to estimate the prevalence, trends, and factors associated with hypertensive crisis at the population level. However, this study holds some limitations that deserve to be discussed. Firstly, due to the cross-sectional nature of the ENDES, it is not possible to determine causality, but only the association between variables. Furthermore, reverse causality cannot be ruled out, as is the case with smoking and alcohol use in unadjusted models. Secondly, it was not possible to obtain information on organ damage during episodes of hypertensive crisis to differentiate urgency and emergency events, as ENDES does not collect clinical or laboratory parameters. Nevertheless, our findings are relevant to understand the epidemiology of hypertensive crisis in Peru. Thirdly, some variables were collected by self-report (smoking, alcohol use, or type 2 diabetes), introducing the possibility of recall bias. Also, during 2020 and 2021, data were mainly collected by telephone rather than the usual face-to-face approach. Although this could affect the results, there was no difference in the distribution of variables during the timeframe studied (data not shown). However, the sample size was small, especially in 2020, due to the restriction of the COVID-19 pandemic. Fifth, although the missing values were low, the lack of data on two variables may affect the results. This is the case for educational level, but especially for type 2 diabetes, which reach around 12% of missing data. Finally, the outcome variable was measured with an automatic device that uses only two readings instead of the three suggested by international guidelines ^15^; however, this simplified method does not seem to affect the results and is associated with low rates of missed cases ^33^.

## Conclusions

Around 1.5% of adult subjects, but 5% of the cases with hypertension, had a hypertensive crisis. The prevalence of hypertensive crises has decreased over time, despite the increase in hypertension rates. Sex, age, living in an urban area, obesity, and type 2 diabetes were positively associated with hypertensive crisis, while educational level, socioeconomic status, and living in high altitude were inversely associated. It is necessary to improve strategies for diagnosing, treating, and controlling hypertension, especially hypertensive crises.

## References

[1] GBD 2017 Risk Factors Collaborators (2018). Global, regional, and national comparative risk assessment of 84 behavioural, environmental and occupational, and metabolic risks or clusters of risks for 195 countries and territories, 1990-2017: a systematic analysis for the Global Burden of Disease Study 2017.. Lancet.

[2] Angell SY, De Cock KM, Frieden TR (2015). A public health approach to global management of hypertension. Lancet.

[3] NCD Risk Factor Collaboration (2021). Worldwide trends in hypertension prevalence and progress in treatment and control from 1990 to 2019: a pooled analysis of 1201 population-representative studies with 104 million participants.. Lancet.

[4] Task Force of the Latin American Society of Hypertension (2017). Guidelines on the management of arterial hypertension and related comorbidities in Latin America. J Hypertens.

[5] Ruiz-Alejos A, Carrillo-Larco RM, Bernabé-Ortiz A (2021). Prevalence and incidence of arterial hypertension in Peru a systematic review and meta-analysis. Rev Peru Med Exp Salud Pública.

[6] Villarreal-Zegarra D, Carrillo-Larco RM, Bernabe-Ortiz A (2021). Short-term trends in the prevalence, awareness, treatment, and control of arterial hypertension in Peru. J Hum Hypertens.

[7] Williams B, Mancia G, Spiering W, Rosei EA, Azizi M, Burnier M (2018). 2018 ESC/ESH guidelines for the management of arterial hypertension. Eur Heart J.

[8] Whelton PK, Carey RM, Aronow WS, Casey DE, Collins KJ, Himmelfarb CD (2018). 2017 ACC/AHA/AAPA/ABC/ACPM/AGS/APhA/ASH/ASPC/NMA/PCNA Guideline for the Prevention, Detection, Evaluation, and Management of High Blood Pressure in Adults: a report of the American College of Cardiology/American Heart Association Task Force on Clinical Practice Guidelines.. Hypertension.

[9] Varounis C, Katsi V, Nihoyannopoulos P, Lekakis J, Tousoulis D (2016). Cardiovascular hypertensive crisis recent evidence and review of the literature. Front Cardiovasc Med.

[10] Muiesan ML, Salvetti M, Amadoro V, di Somma S, Perlini S, Semplicini A (2015). An update on hypertensive emergencies and urgencies. J Cardiovasc Med.

[11] Astarita A, Covella M, Vallelonga F, Cesareo M, Totaro S, Ventre L (2020). Hypertensive emergencies and urgencies in emergency departments a systematic review and meta-analysis. J Hypertens.

[12] Pinna G, Pascale C, Fornengo P, Arras S, Piras C, Panzarasa P (2014). Hospital admissions for hypertensive crisis in the emergency departments a large multicenter Italian study. PLoS One.

[13] Instituto Nacional de Estadística e Informática (2022). Encuesta Demográfica y de Salud Familiar - ENDES 2022.

[14] Campos-Sánchez M, Ricaldi-Sueldo R, Miranda-Cuadros M (2011). Design of the National Surveillance of Nutritional Indicators (MONIN), Peru 2007-2010. Rev Per Med Exp Salud Pública.

[15] Chobanian AV, Bakris GL, Black HR, Cushman WC, Green LA, Izzo JL (2003). The Seventh Report of the Joint National Committee on Prevention, Detection, Evaluation, and Treatment of High Blood Pressure: the JNC 7 report.. JAMA.

[16] United Stated Agency for International Development Wealth Index Construction..

[17] West BT, Berglund P, Heeringa SG (2008). A closer examination of subpopulation analysis of complex-sample survey data. Stata J.

[18] Rao JN, Scott AJ (1992). A simple method for the analysis of clustered binary data. Biometrics.

[19] Pierin AMG, Flórido CF, Santos JD (2019). Hypertensive crisis clinical characteristics of patients with hypertensive urgency, emergency and pseudocrisis at a public emergency department. Einstein (São Paulo).

[20] Reis KG, Wilson R, Kalokola F, Wajanga B, Lee M-H, Safford M (2020). Hypertensive urgency in Tanzanian adults: a 1-year prospective study.. Am J Hypertens.

[21] Singh JN, Nguyen T, Kerndt CC, Dhamoon AS (2023). Physiology, blood pressure age related changes.

[22] Caligiuri SP, Austria JA, Pierce GN (2017). Alarming prevalence of emergency hypertension levels in the general public identified by a hypertension awareness campaign. Am J Hypertens.

[23] Sharma K, Mathews EP, Newton F (2021). Hypertensive emergencies a review. Am J Nurs.

[24] Alley WD, Copelin IE (2023). Hypertensive urgency.

[25] van den Born B-JH, Lip GYH, Brguljan-Hitij J, Cremer A, Segura J, Morales E (2019). ESC Council on Hypertension position document on the management of hypertensive emergencies.. Eur Heart J Cardiovasc Pharmacother.

[26] Vilela-Martin JF, Vaz-de-Melo RO, Kuniyoshi CH, Abdo AN, Yugar-Toledo JC (2011). Hypertensive crisis clinical-epidemiological profile. Hypertens Res.

[27] Ruilope LM, Chagas AC, Brandão AA, Gómez-Berroterrán R, Alcalá JJA, Paris JV (2017). Hypertension in Latin America current perspectives on trends and characteristics. Hipertens Riesgo Vasc.

[28] Reckelhoff JF (2018). Gender differences in hypertension. Curr Opin Nephrol Hypertens.

[29] Shao PJ, Sawe HR, Murray BL, Mfinanga JA, Mwafongo V, Runyon MS (2018). Profile of patients with hypertensive urgency and emergency presenting to an urban emergency department of a tertiary referral hospital in Tanzania. BMC Cardiovasc Disord.

[30] Fiório CE, Cesar CLG, Alves M, Goldbaum M (2020). Prevalence of hypertension in adults in the city of São Paulo and associated factors. Rev Bras Epidemiol.

[31] Siddiqi TJ, Usman MS, Rashid AM, Javaid SS, Ahmed A, Clark D (2023). Clinical outcomes in hypertensive emergency: a systematic review and meta-analysis.. J Am Heart Assoc.

[32] Oficina General de Planeamiento. Presupuesto y Modernización del Sector Salud. Ministerio de Salud (2021). Diagnóstico de brechas de infrestructura y equipamiento del sector salud.

[33] Carrillo-Larco RM, Guzman-Vilca WC, Neupane D (2022). Simplified hypertension screening methods across 60 countries an observational study. PLoS Med.

